# Time to initiation of antenatal care and its predictors among pregnant women in Ethiopia: Cox-gamma shared frailty model

**DOI:** 10.1371/journal.pone.0246349

**Published:** 2021-02-05

**Authors:** Reta Dewau, Amare Muche, Zinabu Fentaw, Melaku Yalew, Gedamnesh Bitew, Erkihun Tadesse Amsalu, Mastewal Arefaynie, Asnakew Molla Mekonen

**Affiliations:** 1 Department of Epidemiology and Biostatistics, School of Public Health, College of Medicine and Health Sciences, Wollo University, Dessie, Ethiopia; 2 Department of Reproductive and Family Health, School of Public Health, College of Medicine and Health Sciences, Wollo University, Dessie, Ethiopia; 3 Department of Health Service Management, School of Public Health, College of Medicine and Health Sciences, Wollo University, Dessie, Ethiopia; Ohio University College of Health Sciences and Professions, UNITED STATES

## Abstract

**Background:**

Timely initiating antenatal care (ANC) is crucial in the countries that have high maternal morbidity and mortality. However, in developing countries including Ethiopia, pregnant mother’s time to initiate antenatal care was not well-studied. Therefore, this study aimed to assess time to first ANC and its predictors among pregnant women in Ethiopia.

**Methods:**

A community-based cross-sectional study was conducted among 7,543 pregnant women in Ethiopia using the Ethiopian Demographic Health Survey (EDHS), 2016 data. A two-stage stratified cluster sampling was employed. The Kaplan-Meier (KM) method was used to estimate time to first antenatal care visit. Cox-gamma shared frailty model was applied to determine predictors. Adjusted Hazard Ratio (AHR) with 95% confidence interval was reported as the effect size. Model adequacy was assessed by using the Cox-Snell residual plot. Statistical significance was considered at p value <0.05. For data management and analysis Stata 14 was used.

**Results:**

The median time to first ANC was 5 months with IQR (3,-). The independent predictors of time to first ANC visit were primary education [AHR: 1.24 (95%CI, 1.13–1.35)], secondary education [AHR: 1.28(95% CI, 1.11–1.47)], higher education [AHR: 1.43 (1.19–1.72)] as compared to women with no formal education. Having media exposure [AHR: 1.13 (95% CI, 1.03–1.24)], early initiation of ANC increases by 25% [AHR: 1.25 (95% CI, 1.12–1.40)] in poorer, 32% [AHR: 1.32 (95% CI, 1.17–1.49)] in middle, 37% [AHR: 1.37 (95% CI, 1.20–1.56)] in richer and 41% [AHR: 1.41 (95%CI, 1.1.19–1.67)] in richest households as compared to poorest household wealth index. Living in city administration, media exposure and community women literacy were also enabler factors, while, long distance from health facility and nomadic region residency were hindering factors of early ANC visit.

**Conclusions:**

The current study revealed that women’s time to first antenatal care visit was by far late in Ethiopia as compared to the world health organization recommendation (WHO). The predictors of time to first ANC visit were education status of women, having media exposure, level of household wealth index, community women literacy ad distance to health facility. It is vital that maternal and child health policies and strategies better to be directed at women development and also designing and applying interventions that intended to increase timely initiation ANC among pregnant-women. Researchers also recommended conducting studies using a stronger design like a cohort to establish temporality and reduce biases.

## Introduction

Maternal mortality ratio (MMR), the number of maternal deaths per 100 000 live births, was estimated at 216 globally and almost all (95%) happened in developing countries [1], 412 in Ethiopia [2].

The care given to the mother during preconception, pregnancy, delivery and after delivery is important for the well-being of the mother and her unborn fetus [3]. All pregnant ladies are recommended to visit their first antenatal check-up in the first trimester to identify and manage any medical complications as well as to screen them for any risk factors that may affect the progress and outcomes of their pregnancy [3, 4].

Timely initiation of the ANC is important in countries that have high maternal morbidity and mortality to reduce maternal morbidity and mortality [5]. In developing countries, predominantly in sub-Saharan Africa maternal morbidity and mortality were high. However, most women initiated first ANC at the later trimesters of the pregnancy [3, 5].

Numerous studies had linked timely, and high-quality antenatal care service to improve maternal and newborn health outcome [3, 6]. Early initiation of ANC is broadly recognized as an important gateway for screening and early identification of pregnancy-related complications such as pregnancy induced hypertension, anemia, HIV and gestational diabetes [7]. It also exposes pregnant women to counseling and education about their own and unborn fetus health by providing adequate time. Pregnant women who attended antenatal care at the later trimester compared to the recommended schedules were at increased risks of maternal and child mortality [8].

The mean gestational age at first antenatal care attendance was 22.6±5.7 weeks in northern Uganda [9], 5 ± 1.5 months in Minch District [10], 15.9 (SD 3.7) weeks in Dilla Town [11], 4.5 months in Gondar Town [12], the median duration of pregnancy at the first visit was 5 month in Central Zone, Tigray [13] Ethiopia, and the mean gestational age at booking was 18.3 weeks in Southern Nigeria [14]. Regardless of the time of first ANC, only 62% of pregnant women aged 15–49 years were received antenatal care from a skilled provider [2].

Predictors of timing of first antenatal care follow up from different literatures were residence [11, 15, 16], household wealth [10, 11, 17–19], Education [4, 10, 11, 14, 17, 20–23], media exposure [4, 13, 18], marital status [24], maternal age [12, 25], working status [23, 26], parity [13, 14, 21, 26] husband education [9, 14], husband occupation [22, 23], distance from health facility [9, 16, 27, 28], region [29, 30] and religion [5, 24].

Despite various studies on ANC follow-up were conducted in Ethiopia [2, 10, 20, 25], the exact time of the first antenatal visit was not studied so far. Furthermore, most of the studies are limited to specific districts to be representative at the national level. Therefore, this study was designed to assess the time to first antenatal care and its predictors among pregnant women in Ethiopia at the national level. So, it will have paramount importance for enhancement of new born, pregnant women, and family health and to scale up knowledge on reduction of maternal and child morbidity and mortality in the society. It will also guide policy makers and program planners on the reduction of maternal and child mortality by considering time in addition to the number of ANC visits at pregnancy.

## Methods

### Study design, area, and period

A community-based cross-sectional study was conducted among pregnant women in Ethiopia. The study was conducted from January 18-June 27, 2016 [2].

### Study participants

The study included all pregnant women (15–49 years) found in the selected clusters of the 2016 EDHS data collection period. The study was conducted by taking pregnant women in place of the source population; pregnant women living in selected clusters as the study population and those found in 2016, EDHS enumeration areas at least one night before data collection as per the Sample population [2].

### Sample size determination and sampling technique

**Fig 1:**
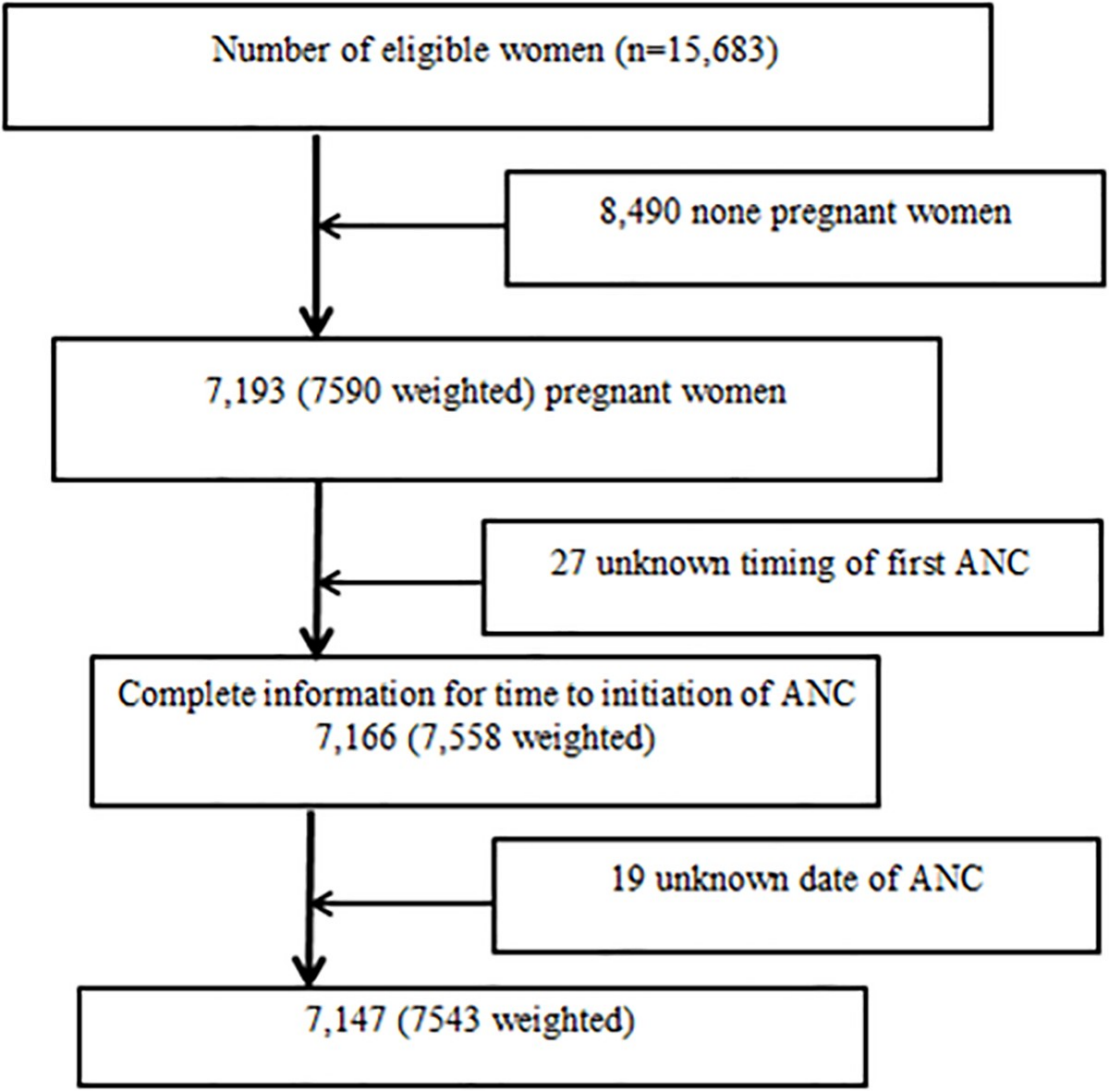
Sample size and sampling procedure to reach the final sample size in 2016 EDHS.

The 2016 EDHS used a stratified two-stage cluster sampling design and census enumeration areas (EAs) were the sampling units for the first stage. The sample included 645 EAs, 202 in urban areas and 443 in rural areas with probability proportional to EA size and with independent selection in each sampling stratum. In the second stage of selection, a fixed number of 28 households per cluster were selected with an equal probability of systematic selection from the newly created household listing. A total of 18,008 households were selected for the sample, of which 17,067 were occupied. In the interviewed households, 16,583 eligible women were identified for individual interviews and 15,683 women were interviewed [2]. For current study 7,543 (weighted) pregnant women were included. Pregnant women who didn’t remember their first antenatal care visit were excluded (Fig 1).

### Variable measurement

The dependent variable in the current study was the time to the initiation of antenatal care in months. The time variable in this study was the time from pregnancy to the first antenatal care visit. Time component was taken first antenatal care in month (s) if she had follow-up, otherwise the gestational age of the corresponding pregnancy was considered. The event of interest considered as success (event = 1) if women had antenatal care and (event = 0) otherwise. The independent variables included, socio-demographic factors such Socio-demographic factors such as age (ordinal categorical variable with categories ‘15–19’, ‘20–24’, ‘25–29’, ‘30–34’ ‘35–39’ and “>40 years”), and religion (nominal categorical variable with categories ‘orthodox’, ‘Muslim’, ‘protestant ‘and ‘others’), marital status (nominal categorical variable with “not married”, “married”, and”Widowed and others”) and spousal age difference (ordinal variable “less than 5 years”, “5-10years” and “greater than 10 years”); socio-economic factors such as education level (ordinal categorical variable with categories ‘no education’, ‘primary education’, ‘secondary education’, and ‘higher education’), respondents occupational status (nominal categorical variable with “house wives”, “agricultural” or “none agricultural”), wealth index (ordinal categorical variables with”poorest”,”poorer”,”middle”,”richer” and “richest”), husband’s education as women, husband occupation (nominal categorical variable with “not working”, “agriculture” and “nonagricultural”), distance from health facility (nominal categorical variable with “distance is problem” and “distance is not problem”) and Mass media exposure (nominal categorical variable with “yes” and “no”); obstetric factors such as parity (ordinal variable with “1”, “2–3”, and “4+”) age at pregnancy (ordinal variable with “below 20 years”, “20–24”, “25–34” and “older than 35 years”) and Community level factors like region (nominal categorical variables with “Agrarian”, “Pastoralist” and “Urban”) and residence (nominal categorical variables with “urban” and “rural”) community level women literacy and husband literacy (nominal categorical variables with “illiterate” and “at least primary”), community media access (nominal categorical variables with “not accessed” and “accessed”) and community poverty level (nominal categorical variables with “in poverty level” and “above poverty”).

### Operational definitions

#### Time

Time was measured in month(s) from date of pregnancy to first ANC booking for women’s having at least one ANC visit and their current gestational age otherwise.

#### Event

Event was considered happened if the pregnant women had at least one ANC ad considered censored otherwise.

#### Media exposure

Those who responded at least once a week for read a newspaper, listened to the radio, or watched television are considered to be regularly exposed to media and other considered as had no media exposure [2].

#### Region

Regions (Amhara, harari, Oromia, SNNP and Tigray) whose livelihood mainly based on agriculture considered and with better distribution of health facilities classified as agrarian, regions whose livelihood based on mainly nomadism (Somali, Benshangul-Gumuz, Gambela and Afar) were with less access of Healthcare services considered as pastoralist or emerging regions and urban regions (city administration) those livelihood based on employment and trade (Addis Ababa and Dire Dawa) [31, 32].

#### Community media exposure

Community considered as exposed to media if more than 50% of the community exposed to media and otherwise unexposed.

#### Community women literacy

Community considered as literate if at least 50% of women in the community attained at least primary education and illiterate if women in the community had no education or only less than half proportion of women in the community educated.

#### Community husband literacy

Community husband considered as literate if at least 50% of husband in the community attained at least primary education and illiterate if husband in the community had no education or only less than half proportion of the husband in the community only educated.

### Data source and analysis

For this study secondary data from the 2016 EDHS was used. The data set downloaded from the website https://dhsprogram.com after approval letter for use had been obtained from the measure DHS. Variables were extracted from the EDHS 2016 kids and individual women’s data set using a data extraction tool. After data management, cleaning and weighting descriptive measures such as median, percentage, graphs, and frequency tables were used to characterize the study population. Time to first ANC visit was estimated using the Kaplan-Meier (K-M) method. The log-rank test was applied to compare survival time difference between groups of categorical variables with outcome of interest. A likelihood ratio test for a variance of frailty *θ* = 0 was checked and a statistical significant with p-value of <0.05 for cox-gamma shared frailty model were considered the frailty component contributes to the model and suggested presence of a within-cluster correlation. Cox gamma shared frailty model was modeled by taking enumeration areas/clusters as a random effect to identify predictors of time to first antenatal care booking among pregnant women in Ethiopia. Model adequacy was checked using Cox-Snell residuals subjective evaluation Stata 14.0/SE was used for the data management and analysis.

In statistical terms, a frailty model is a random effect model for time-to-event data, where the random effect (the frailty) has a multiplicative effect on the baseline hazard function [33]. In shared frailty study, the survival experience of individuals from the same cluster may be more similar than that for individuals from different clusters. Thus it is responsible for creating dependence between event times in a cluster. This dependence is always positive in shared frailty models. Conditional on the random effect, called the frailty denoted by *ui*, the survival times in cluster i (1 ≤ i ≤ n) are assumed to be independent and the proportional hazard frailty model assumes:

hij(t|xij,ui)=ho(t)exp(βxij+ui))

where *i* indicates the *i*^th^ cluster and j indicates the jth individual for the *i*^th^ cluster, *ho*(*t*) is the baseline hazard function, *ui* the random term of all the subjects in cluster *i*, *xij* the vector of covariates for subject j in cluster *i* and β the vector of regression coefficients.

If we tried to estimate each subject’s frailty (*ui*), then there would be more parameters to estimate than observations in the dataset and the model would be over-parameterized. Rather, the variance of the frailty is estimated. The gamma distribution is a two-parameter distribution. Because the mean is set at 1, we need only estimate its variance (θ) to fully specify the frailty distribution.

The associations within group members are measured by Kendall's Tau, which is given by

$$\tau =\frac{\theta }{\theta +2}\;where\;\tau \;\varepsilon (0,1)$$

### Ethical considerations

As the study was secondary data analysis, the dataset were downloaded from the website https://dhsprogram.com after legal registration and approval letter was obtained from the measure DHS. The data were used only for this study and it was not passed to other researchers. All data were treated as confidential and no personal or household identifiers were used in the survey. The detailed information on ethical issues was published within the EDHS report.

## Results

### Socio-demographic characteristics

Out of 7,543 (weighted) pregnant women included in the study almost about 2,158 (28.60%) were in the age group 25–29 years and 4.48% were less than 20 years. Most of the pregnant women in this study (93.67%) were married. Regarding to religious affiliation orthodox Muslim and protestant worshipers were 37.89%, 37.27% and 21.78% respectively. Majority of pregnant women (63.03%) in the current study had no formal education. and only 8.87% women were attained secondary and higher education level. More than half (53.73%) of the participants were housewives. Only below a fifth (19.60%) women were exposed to broad cast media. Education statuses of the pregnant women’s husbands were almost similar to the corresponding pregnant women. Almost two third (64.75) of respondents husband were agrarian. Regarding to household wealth status more than two-third (64.44%) of pregnant women were middle and below middle wealth level. Majority of women (58.10%) considered the distance of health facility from their dwelling was a problem.

### Community-level factors

Of all participants in the study 87.25% were rural residents. Majority (92.24%) of respondents lived in agrarian regions and only 4.48% and 3.28% of them were from pastoralist ad urban regions of Ethiopia respectively. Broadcast media access at community level was only 22.45%, women literacy at community level at least for primary education was 28.58% for women and husband literacy at community level at least for primary education was 45.99%. Almost half women (49.62%) at community level were below poverty level (Table 1).

**Table 1:** Socio-demographic, obstetric and community-level characteristics of women in Ethiopia, EDHS 2016.

Variables	Categories	Number (%)	Variables	Categories	Number (%)
First antenatal care	Yes	4,725 (62.64)	Husband education	No formal education	3,364 (47.61)
	No	2,818 (37.36)		Primary education	2,719 (38.47)
Age(years)	15–19	338 (4.48)		Secondary education	610 (8.64)
	20–24	1,450 (19.22)		Higher education	372 (5.27)
	25–29	2,158 (28.60)	Husband occupation	Not working	525 (7.43)
	30–34	1,651 (21.90)		Agriculture	4,575 (64.75)
	35–39	1,201 (15.92)		Non agricultural	1,965 (27.81)
	≥40	745 (9.87)	Distance from HF	Problem	4,382 (58.10)
				No problem	3,161 (41.90)
Marital status	Not married	56(0.74)		Problem	4,382 (58.10)
	Married	7,066 (93.67)	Age at pregnancy	<20 years	698 (9.25)
	Widowed	422 (5.59)		20–24	1,770 (23.47)
Spousal age difference	<5 years	3,259 (46.12)		25–34	3,524 (46.72)
	5-10years	2,361 (33.41)		≥35	1,551 (20.56)
	>10 years	1,446 (20.47)	Parity	1	1,419 (18.82)
Religion	Orthodox	2,858 (37.89)		2–3	2772 (30.12)
	Muslim	2,812 (37.27)		4+	3852 (51.06)
	Protestant	1,643 (21.78)	Community media exposure	Not accessed	5,850 (77.55)
	Others	230 (3.05)		Accessed	1,693 (22.45)
Women education level	No	4,755 (63.03)	Community poverty	In poverty level	3,743 (49.62)
	Primary	2,142 (28.39)		Above poverty	3,800 (50.38)
	Secondary	418 (5.53)	Community women literacy	Below primary	5,388(71.42)
	Higher	230 (3.04)		At least primary	2,155 (28.58)
Women occupation	Housewives	4,053 (53.73)	Community husband literacy	Below primary	4,073 (54.01)
				At least primary	3,459 (45.99)
	Agriculture employ	1,735 (23.00)		Below primary	4,073 (54.01)
	Nonagricultural	1,755 (23.70)	Residence	Urban	962 (12.75)
Media exposure	Yes	1,478 (19.60)		Rural	6,581 (87.25)
	No	6,065 (80.40)	Region	Agrarian	6,958 (92.24)
Wealth index	Poorest	1,641 (21.75)		Nomadic	338 (4.48)
	Poorer	1,650 (21.87)		City administration	248 (3.28)
	Middle	1,572 (20.84)			
	Richer	1,422 (18.85)			
	Richest	1,259(16.69)			

### Time to first antenatal care among pregnant women in Ethiopia

**Fig 2:**
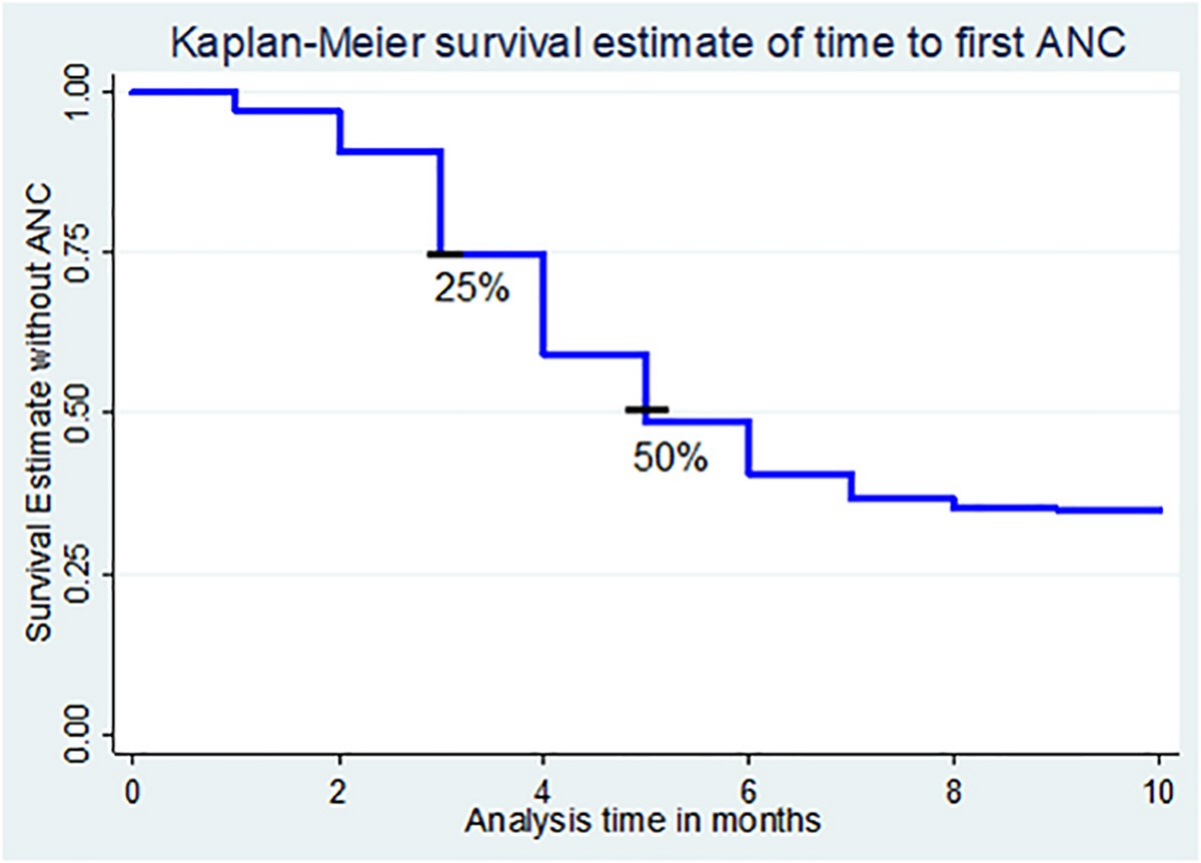
Kaplan-Meier survival estimate of pregnant women before they were attending ANC, Ethiopia, 2016 EDHS.

In the current study participants were assessed retrospectively for 44,139 person-months. The overall at least one antenatal care follow up was 62.64% and the median time at first antenatal care in months was 5± (IQR: 3, -) and only less than a quarter pregnant women were booked for ANC within first trimester (Fig 2).

The first antenatal care was as early as 3 months ± (IQR: 1, 4) among women with higher education, urban residence 3 months ± (IQR: 2, 5) and most urban regions 3 months ± (IQR: 1, 5) months. It was as late as at 8 months (IQR: 3,-) for mothers in age group ≥40 years and women with husband had no formal education, and 7 months (IQR: 3,-) women no formal education level, women reported that distance from health facility is problem, parity of 5 and above children, women in poverty level community, bellow primary women and husband community literacy with corresponding log-rank test significance value (Table 2).

**Table 2:** Time to first antenatal care (in months) among pregnant women in Ethiopia EDHS, 2016.

Variable		Event (first antenatal care)		median±IQR	log-rank test chi-square	p-value
		Yes (%)	No (%)			
Socio-demographic factors						
Age(years)	15–19	251 (74.12)	87 (25.88)	5±(2,-)	96.54	0.001
	20–24	970 (66.89)	480(33.11)	5±(2,-)		
	25–29	1,447 (67.07)	711(32.93)	5±(2,-)		
	30–34	1,032 (62.48)	620(37.52)	5±(3,-)		
	35–39	655 (54.51)	546(45.49)	6±(3,-)		
	≥40	370 (49.76)	374(50.24)	8±(3,-)		
Marital status	Not married	40 (71.27)	16(28.73)	5±(2,-)	4.87	0.088
	Married	4,438 (62.81)	2,628(37.2)	5±(3,-)		
	Widowed	247 (58.64)	175(41.36)	5±(2,-)		
Spousal age difference	<5 years	2,085 (63.98)	1,174(36.02)	5±(2,-)	24.11	0.001
	5-10years	1,489(63.05)	872(36.95)	5±(2,-)		
	>10 years	864 (59.78)	582 (40.22)	6±(3,-)		
Religion	Orthodox	2,006 (70.19)	852 (29.81)	4(2,7)	314.50	0.001
	Muslim	1561(55.50)	1,251(44.50)	6±(3,-)		
	Protestant	1041(63.35)	602(36.65)	6±(3,-)		
	Others	118(51.03)	113(48.97)	-		
Socio-economic factors						
Women education level	No	2,544 (2,543)	2,211(46.50)	7± (3,-)	1313.32	0.001
	Primary	1569(73.26)	573(26.74)	4± (2,6)		
	Secondary	386(92.33)	32(7.67)	4± (2,5)		
	Higher	226(98.89)	3(1.11)	3± (1,4)		
Women occupation	Housewives	2,396(59.11)	1,657(40.59)	6±(3,-)	240.31	0.001
	Agriculture employ	1,069(61.62)	666(38.38)	6±(3,-)		
	Non agricultural	1,260(71.80)	495(28.20)	4± (2,7)		
Media exposure	Yes	1,194 (80.78)	284(19.22)	4± (2,5)	820.22	0.001
	No	3,531 (58.22)	2,534(41.78)	6±(3,-)		
Wealth index	Poorest	784(47.77)	857(52.23)	-	1624.99	0.001
	Poorer	931(56.44)	719(43.56)	6± (3,-)		
	Middle	980(62.33)	592 (37.67)	5± (3,-)		
	Richer	962 (67.65)	462 (32.35)	5± (3,-)		
	Richest	1,068(84.86)	191 (15.14)	4 ± (2,5)		
Husband education	No	1,792(53.25)	1,572 (46.75)	8± (3,-)	862.58	0.001
	Primary	1,803 (66.34)	915 (33.66)	5± (3,-)		
	Secondary	510(83.66)	100 (16.34)	4± (2,6)		
	Higher	332 (89.20)	40 (10.80)	4± (2,5)		
Husband occupation	Not working	256 (48.79)	269 (51.21)	-	525.47	0.001
	Agriculture	2,655 (58.02)	1,920 (41.98)	6± (3,-)		
	Non agricultural	1527 (77.70)	438(22.30)	4± (2,6)		
Distance from HF	Problem	2,378(54.25)	2,005 (45.75)	7± (3,-)	461.75	0.001
	No problem	2,348(74.27)	813 (25.73)	4± (2,7)		
Obstetric factors						
Age at pregnancy	<20 years	471 (67.53)	227 (32.47)	5± (3,-)	76.39	0.001
	20–24	1,191(67.31)	579 (32.69)	5± (2,-)		
	25–34	2,252(63.89)	1273(36.11)	5± (2,-)		
	≥35	811 (52.27)	740 (47.73)	6± (3,-)		
Parity	1	1,108(78.03)	311(21.97)	4± (2,7)	428.81	0.001
	2–3	1,531 (67.38)	741 (32.62)	5± (2,-)		
	4 and above	2087 (54.17)	1765 (45.83)	7± (3,-)		
Community level factors						
Community media exposure	Not accessed	3,394 (58.02)	2,456 (41.98)	6± (3,-)	940.93	0.001
	Accessed	1,331(78.59)	362 (21.41)	4± (2,5)		
Community poverty	In poverty level	2,046(54.67)	1,697(45.33)	7± (3,-)	769.42	0.001
	Above poverty	26.79 (70.49)	1121 (29.51)	4± (2,6)		
Community women literacy	Illiterate	2,980(55.32)	2,407(44.68)	7± (3,-)	965.02	0.001
	At least primary	1745 (80.93)	411 (19.07)	4± (2,6)		
Community husband literacy	Illiterate	2,292 (56.27)	1,781(43.73)	7± (3,-)	619.13	0.001
	At least primary	2,431(70.10)	1,037(29.90)	4± (2,7)		
Residence	Urban	868 (90.26)	94 (9.74)	3± (2,5)	1299.37	0.001
	Rural	3,857 (58.60)	2,724 (41.40)	6± (3,-)		
Region	Agrarian	4,339 (62.36)	2619 (37.64)	5± (3,-)	929.38	0.001
	Pastoralist	153 (45.27)	185 (54.73)	-		
	Urban	233 (94.05)	15 (5.95)	3± (1,5)		
**Total**		4,725(62.64)	2,818 (37.36)	5± (3,-)		

HF health facility “-“unknown third quartile.

### Predictors of time to first antenatal care among pregnant women in Ethiopia

Holding other factors constant and keeping women in the same cluster early initiation of antenatal care reduced by 24% among Protestants (AHR: 0.76, 95% CI, 0.68–0.86) and 39% among others (AHR: 0.61, 0.46–0.81) as compared to orthodox religious followers (Table 3).

**Table 3:** Cox-gamma shared frailty multivariable analysis of time to first antenatal care among pregnant women in Ethiopia.

Variable	Categories	Model 1 Empty	Model 2 Community	Model 3 Individual	Model 4 Individual & community
		CHR (95% CI)	AHR (95% CI)	AHR (95% CI)	AHR (95% CI)
Age(years)	15–19			1.05(0.90–1.24)	1.19 (0.98–1.45)
	20–24			1	1
	25–29			1.16(1.05–1.28)**	1.07 (0.93–1.22)
	30–34			1.18(1.04–1.33)**	1.06 (0.90–1.25)
	35–39			1.11(0.97–1.28)	0.96 (0.7–1.18)
	≥40			0.97(0.82–1.15)	0.83 (0.64–1.08)
Spousal age difference	<5 years			1	1
	5-10years			1.01(0.94–1.09)	1.02(0.95–1.10)
	>10 years			1.03(0.95–1.12)	1.05(0.96–1.14)
Religion	orthodox			1	1
	Muslim			0.89(0.80–0.98)**	0.96(0.86–1.07)
	protestant			0.76 (0.68–0.86)**	0.76 (0.68–0.86)**
	others			0.61(0.46–0.81)**	0.61 (0.46–0.81)**
Women education level	No			1	1
	primary			1.30(1.20–1.41)**	1.24(1.13–1.35)**
	Secondary			1.39(1.21–1.59)**	1.28(1.11–1.47)**
	higher			1.60(1.33–1.92)**	1.43(1.19–1.72)**
Women occupation	Housewives			1	1
	agriculture employ			1.04(0.94–1.14)	1.04 (0.94–1.14)
	nonagricultural			1.05(0.96–1.13)	1.05 (0.96–1.14)
Media exposure	Yes			1.16(1.06–1.27)**	1.13(1.03–1.24)**
	no			1	1
Wealth index	Poorest			1	1
	Poorer			1.34(1.20–1.49)**	1.25(1.12–1.40)**
	Middle			1.43(1.28–1.61)**	1.32(1.17–1.49)**
	Richer			1.50(1.33–1.70)**	1.37(1.20–1.56)**
	Richest			1.94(1.70–2.22)**	1.41(1.19–1.67)**
Husband education	No			1	1
	primary			1.24(1.15–1.35)**	1.21 (1.12–1.32)**
	Secondary			1.32(1.17–1.49)**	1.26 (1.12–1.43)**
	higher			1.34(1.16–1.55)**	1.33 (1.15–1.54)**
Husband occupation	not working			1	1
	agriculture			1.07(0.94–1.22)	1.05 (0.92–1.20)
	non_ agriculture			1.22(1.07–1.39)**	1.19(1.04–1.35)**
Distance from HF	problem			0.86 (0.80–0.94)**	0.87 (0.81–0.94)**
	no problem			1	1
Age at pregnancy	<20 years			1	1
	20–24			1.21 (1.03–1.42)	1.19 (1.01–1.39)**
	25–34			1.23 (0.99–1.51)	1.18 (0.96–1.45)
	≥35			1.31 (1.01–1.71)	1.24 (0.95–1.62)
Parity	1			1	1
	2–3			0.83 (0.75–0.91)	0.83 (0.76–0.92)**
	4 and above			0.77 (0.68–0.87)	0.80 (0.71–0.91)**
Community media exposure	not accessed		1		1
	accessed		1.17(0.99–1.37)		0.99 (0.85–1.15)
Community poverty	In poverty level		1		1
	Above poverty		1.23(1.09–1.41)		0.96 (0.84–1.09)
Community women literacy	Illiterate		1		1
	At least primary		1.36(1.18–1.57)		1.24 (1.08–1.42)**
Community husband literacy	Illiterate		1		1
	At least primary		1.08(0.94–1.23)		1.01 (0.88–1.14)
Residence	Urban		1		1
	Rural		0.62(0.52–0.74)		0.82 (0.69–0.99)**
Region	Agrarian		1		1
	Pastoralist		0.62 (0.53–0.72		0.71 (0.60–0.84)**
	Urban		1.36(1.18–1.57)		1.33 (1.15–1.55)**
Measures of variation (θ)		0.593148	0.2411574	0.2175911	0.1909605
Kendall’s tau (τ)		0.229	0.108	0.098	0.087
MHR		2.09(2.03–1.14)	1.60(1.52–1.66)	1.56 (1.48–1.63)	1.52 (1.45–1.58)**
log likelihood		-39052.5			-35366.4
R^2^ type statistics		62.32%			

** Significant at ***α*** = 5%, HF health facility.

Control for other factors and having the same frailty early initiation of ANC increases by 24% (AHR: 1.24, 95%CI, 1.13–1.35) women with primary education, 28% (AHR: 1.28, 95%CI, 1.11–1.47) secondary education and 43% (AHR: 1.43, 95%CI, 1.19–1.72) higher education compared to women have no formal education.

Keeping them in the same cluster and adjusting for other factors women having media exposure had 13% (AHR: 1.13, 95%CI, 1.03–1.24) increased early initiation of ANC visit than had no media exposure.

At the same level of predisposition and holding constant other factors women early initiation of ANC increases by 25% (AHR:1.25,95%CI,1.12–1.40) in poorer, 32% (AHR:1.32,95%CI,1.17–1.49) in middle, 37% (AHR:1.37,95%CI, 1.20–1.56) in richer and 41% (AHR:1.41, 95%CI,1.1.19–1.67) in richest households as compared to poorest household.

Early initiation of ANC increased 1.21 times (AHR: 1.21, 95%CI, and 1.12–1.32) among women having husbands of primary education, 1.26 times (AHR: 1.26, 95%CI, 1.12–1.43) secondary education ad 1.33 time (AHR: 1.33, 95%CI, 1.15–1.54) higher education as compared to women having husbands with no formal education in the same level of frailty and making constant other factors.

Women with non-agricultural employed husbands were 19% (AHR: 1.19, 95CI, 1.04–1.39) increased early ANC utilization compared to Women with not working husbands in same cluster and adjusting for other factors.

Women those were reported that the distance of health facility as main problem reduced early utilization of ANC by 13% (AHR: 95%CI, 0.81–0.94) compared to women reported have no distance problem holding constant other factors effect keeping women in the same cluster.

Adjusting for other factors early initiation of ANC was 1.19 times increased (AHR: 95%CI, 1.01–1.39) among 20 to 24 years age at pregnancy than age group less than 20 years at pregnancy.

Holding other factors constant and assuming women in the same cluster women having two to three live children and four and above had hazard of 17% AHR:0.83(95%CI, 0.76–0.92) and 20% AHR: 0.80 (95%CI, 0.71–0.91) delayed initiation of ANC follow up as compared to one parity.

At least primary community women literacy level increase ANC follow up at early in pregnancy by 24%, AHR: 1.24 (95%CI, 1.08–1.42) as compared to illiterate women in community control for other factors and keeping them in the same cluster.

Regarding to place of residence women in rural resident attained ANC 18% AHR: 0.82 (95%CI, 0.69–0.99) later than urban counter parts holding other factors and in the same cluster effect.

Concerning regions of residence women in pastoralist region were 29% AHR: 0.71 (95%CI, 0.60–0.84) reduced early initiation of ANC, whereas urban region women had 33%, AHR: 1.33 (1.15–1.55) higher early pregnancy ages at initiation of ANC as compared to agrarian women adjusting other factors constant and assuming all women in the same cluster.

### Random effect

A likelihood ratio test for a variance of frailty *θ* = 0 yields a highly statistically significant p value of <0.001 for cox gamma shared frailty model (Table 3), suggesting that the frailty component contributes to the model and that there is a within-cluster correlation. The value of shared frailty distribution (θ) is 0.59, 0.24, 0.22 and 0.19 for empty, community factors, individual factors and full models respectively. The dependence within clusters (EAs) was (τ = 23%) before adjusting for predictors and (τ = 8.7%) after adjusting for predictors.

Assuming the majority of women in the cluster to fall within one standard deviation of the norm of the cluster, prior to adjusting for predictor variables the median increase was 2.09 (MHR: 2.09, (95% CI 2.03–1.14) times for women in higher opportunity clusters as compared to women in lower opportunity clusters. Whereas after accounting for predictor factors the median increase in early initiation of ANC among when at higher opportunity cluster was only 1.52 times MHR: 1.52, (95%CI, and 1.45–1.58) as compared to women in the lower opportunity clusters. This difference between null model and full model was almost similar with the overall prediction of the model with the current covariates 62.32% (R^2^ = 0.6232).

### Model adequacy

**Fig 3:**
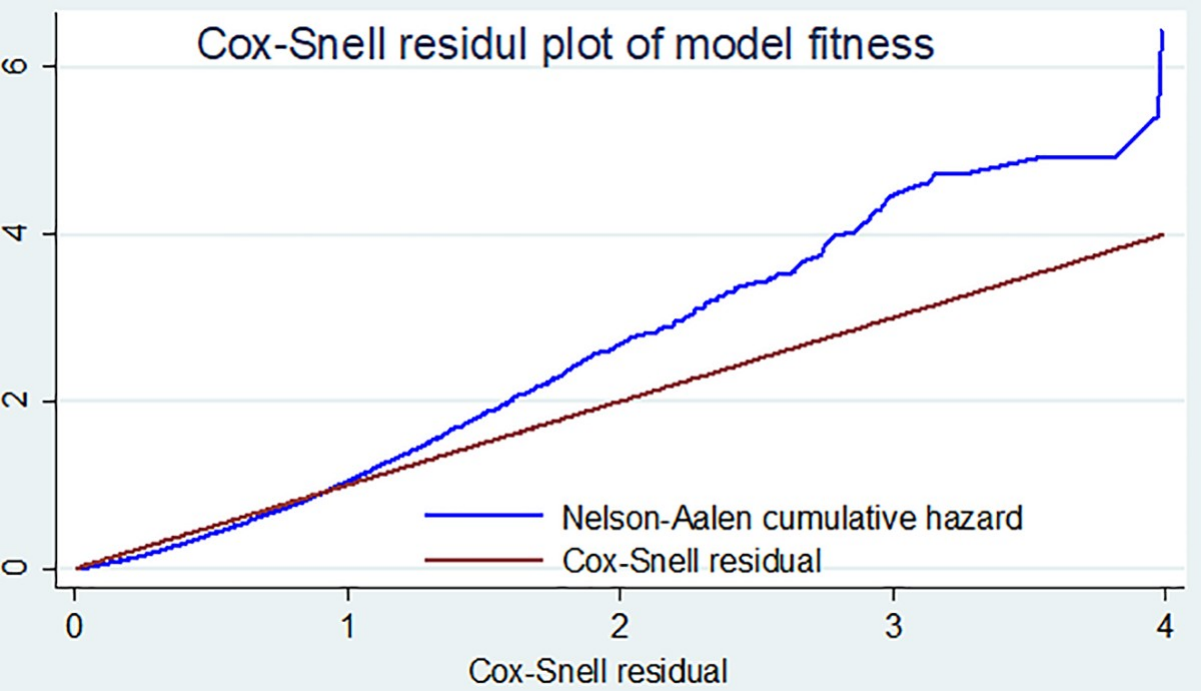
COX-Snell residual plot of time to first ANC and its predictors among pregnant women in Ethiopia, 2016 EDHS.

The Cox-Snell residuals versus the Nelson-Aalen cumulative hazard function were obtained by fitting the cox gamma shared frailty model. The Nelson Aalen cumulative hazard function against the Cox-Snell residuals has a somewhat linear pattern through the origin of the cox gamma shared frailty model. This suggests that the Cox-gamma shared frailty regression model provided the good fit for the time to first ANC initiation data analysis (Fig 3).

Some variability about the 45° line in the right-hand tail is due to reduced effective sample caused by preceding event occurrence and censoring.

## Discussion

Time to initiation of ANC and its predictors were determined using the 2016 Ethiopian Demographic Health Survey data. The overall at least one antenatal care follow up was 62.64% and the median time at first antenatal care in month (s) was 5± (IQR: 3, -) in the current study. Religion, women education, media exposure, household wealth index, husband education, husband occupation, distance from health facility (HF), age at pregnancy, parity from individual factors and community women literacy, residence and region from community factors were the independent predictors of time to first antenatal care.

This result is consistent with findings in Arba Minch Town and Arba Minch District [10], Central Zone, Tigray [13] Ethiopia and in northern Uganda [9]. However, this finding is higher than that in the studies done in Dilla Town [11], Gondar Town [12] and WHO recommendation that states first visit should be within 12 weeks [34]. This variation also could be explained that study participants in these studies were from towns where women had access to education, media exposure and distance from health facility didn’t create significant problem for early booking of antenatal care, which significantly impact the outcome of interest than in the current study. Moreover, this study revealed more than 25% of pregnant women gave birth without visiting health facility for prenatal care.

In this study, women’s educational status was positively and significantly related with early initiation of antenatal care visit compared to women had no formal education. This finding corroborates with study findings in Dilla Town, Southern Ethiopia [11], Debre Markos town, North West Ethiopia [20], in Dilla town south Ethiopia [21], in Nigeria [14, 19] and Nepal [17]. This might be attributed with educated women knowledge on the benefits of ANC utilization that improve, health of mothers and babies through early detection of pregnancy related complications, receiving various preventive and health promotion services [35]. Additionally educated women could have access to mass Medias those advocate the values of timely initiation of ANC and healthcare facilities providing the service. Contrary to these, uneducated women might have limited understanding of ANC services and their importance to their unborn fetus wellbeing and ensure safe delivery.

Regarding to religious affiliation protestant and others (traditional and catholic) worshipers were negatively and significantly linked with early booking for ANC follow up. This finding also supported with results in Zimbabwe [5], To the authors’ knowledge, religion specific determinants of ANC service use have not been elucidated in Ethiopia; however, this might be a reflection of highly organized religious organizations like orthodox church critical role in playing positive health practice compared to others in developing countries in influencing maternal and newborn care services positively [36]. Another reason could be religion-specific differences in education, media access and distance to health facilities, as well as the quality of service provision. Particularly traditionalists reside in rural and remote areas which are hard-to-reach areas for assessing healthcare services including ANC. So, due to these reasons their ANC utilization also may be reduced. This finding was contradict with finding in rural South Africa [24], highly religiously women delayed their ANC. This difference could be due to in our context dominant religious organization (Orthodox Church and Muslim’s mosque) proximity to work with government policies and programs through health developmental army and health extensions workers to improve maternal and child health [36].

Increasing parity was found in this study to predispose to late antenatal care initiation. This finding also supported in other studies [13, 14, 21, 26]. High parity women might have a tendency to depend on their experiences from previous pregnancies and delivery and due to their higher level of experience, so these women might feel more confident during pregnancy and consider antenatal care to be less important. This is especially noteworthy as the grand multiparous women represent a high-risk for adverse outcome in pregnancy [3]. This overconfidence is inappropriate as several studies have continued to document increased and sometimes terrifying difficulties in this group of women [37, 38].

The current study revealed women having educated husband associated with early antenatal care visit. This finding was in agreement with findings in northern Uganda [9], A systematic review and meta- analysis in Ethiopia [15] and in India [18]. Educated husbands may have positive maternal and child health knowledge to encourage their wives to initiate antenatal care at early age of pregnancy. One reason could be also those women have educated husband also will be educated themselves. The combination of these may also determine their residence, economy and access to healthcare knowledge and health services finally which enhance timely antenatal care. Our study indicates the significance of targeting unschooled spouses with health promotion messages, as the majority of mothers who did not attend ANC had no education. This may later lead to timely utilization of ANC service in the long-term.

Consistent with several studies [11, 17–19] the household wealth quintile showed a significant relationship with early initiation of ANC. The challenges to improving the utilization of maternal healthcare services in Ethiopia go beyond improving coverage of the maternal health services, rather demands addressing socioeconomic disparities in accessing maternal health services is vital to resolving problems of maternal health [39]. Women from higher households’ wealth quintiles may have more autonomy, better schooling, and deepened knowledge and self-reliance to decide on their health and unborn baby. So, they seek health care service including timely initiation of antenatal care compared to women in lower household wealth quintile.

Women’s exposure to mass media was positively and significantly related with early ANC booking, and this is consistent with previous studies [4, 13, 18]. Presence of media access is indirectly indicates the relative wealthy household, urban residency, better education level and easy access of healthcare services. The sums of these factors also empower women to have autonomy to engage in healthcare services that improve the health the women and unborn baby including timely commencement of ANC [40, 41].

This research has also shown negative association between distance and early ANC visit. northern Ethiopia [27], Studies in northern uganda [9] a systematic review in Ugandan [16], and Burkina Faso [28] had revealed that ANC utilization affected by distance from health facility, there was a decay effect of the early ANC utilization as distance of the health facility increased from their residence. This distance related delay and poor ANC uptake might be related to the lack of transportation and transportation cost as well as poor knowledge regarding maternal and child health as the majority of them from rural part of the country. So, this finding emphasizes the need of balancing health services inequalities particularly in rural part of the country to achieve maternal and child health goals by using timely ANC as entry point.

Rural women were 0.78 times less likely to be booked early for their first ANC visit than urban dweller women. This finding is supported by a study conducted in Dilla Town, Southern Ethiopia [11], A systematic review and meta- analysis in Ethiopia [15] and Uganda [16]. This could be due to the fact that rural residents are faraway from health facilities, are less educated and more ignorant regardig to ANC. Furthermore this study also showed women with no formal education were 0.70 less likely early booking for ANC visit than women who have higher formal education. This implies accessing health facility in the short radius will increase the early initiation of antenatal care and improve maternal and child health.

Since Ethiopia is spread over a wide geographical area, there are regional discrepancies in the usage of ANC. Particularly pastoralist dominant regions (Somalia and Afar) early prenatal care booking reduced by 29% as compared to agrarian regions. This could be explained that pastoralists’ livelihood is nomadism and they rarely settled in the same area to be easily accessed proper health service. Moreover, majority of these women are uneducated and ignorant to the benefit of early booking for ANC. On the other hand urban dominant regions (Addis Ababa and Dirie Dawa) women were 33% more likely for ANC early booking compared to agrarian regions. The reasons might include the better educational status of women, media exposure, shorter health facility distance and access of transportation as well as overall knowledge of women on the importance of early initiation of ANC. This finding is supported with other findings in Indonesia [29] and in Andhra Pradesh [30]. It is also uncovered that in our study community level women literacy significantly associated with early initiation of ANC. Our study indicates the geographical variations which need to be addressed urgently and aggressively so as to improve maternal and child health.

In addition to fixed effect model the random effect model also contributed in the determination of time to initiation of ANC. Before adjusting for predictor variables the median increase in early initiation of ANC was 2.09 (MHR: 2.09, (95% CI 2.03–1.14) times for women in higher opportunity clusters as compared to women in lower opportunity clusters. Whereas after accounting for predictor factors the median increase in early initiation of ANC among women at higher opportunity cluster was only 1.52 times MHR: 1.52, (95%CI, and 1.45–1.58) as compared to women in the lower opportunity clusters. Meaning that the cluster effect from null model to full model explained with predictors almost by 57% This difference between null model and full model was almost similar with the overall prediction of the model with the current covariates 62.32% (R^2^ = 0.6232). These imply significant disparity of ANC service utilization across clusters which require a context based interventions to tackle the late ANC booking and poor of ANC uptake.

This study was not immune from limitations, primarily presence of missed factors to be investigated on the issue were revealed with higher median hazards ratio (MHR = 1.52) even after adjusting for all community and individual factors as the study is based on secondary data, next as the study is based on cross sectional data temporality between time to antenatal care initiation and predictor factors difficult to establish and finally since data is based on maternal self-recall the data could be predisposed for both recall and social desirability bias. However, these limitations will not prevent generalization of the study to whole reproductive age women in Ethiopia and similar socio-economic level nations of Africa as it was conducted based on nationally representative data which was collected across all the regions of Ethiopia with recognized quality and 95% response rate. Additionally this study was identified both individual and community level factors based on proper statistical analysis method.

This study is significantly important for public health planners and programmers concerning to improve maternal and child health through early pregnancy health care provision by identifying the underlying individual and community factors linked with poor service utilization.

## Conclusions

The current study revealed that women’s time of visiting health facility for first antenatal care was by far late in the study area as compared to world health organization recommendation. The independent predictors, higher wealth quintile, partner education level, urban region residency, media exposure and community women literacy were enabler factors for early ANC initiation and increased parity, long distance from health facility and pastoralist region residency were hindering factors of early ANC booking. It is vital that current and future maternal and child ambitions directed at women socioeconomic empowerment, while also designing and applying multipronged interventions that intended to increase ANC acceptance timely among unwarranted Ethiopian women. Researchers also recommended conducting studies stronger design like cohort to establish temporality with reduced biases.

## Lists of abbreviations and acronyms

AHR: Adjusted Hazard Ratio
ANC: Antenatal Care
EDHS: Ethiopian Demographic Health Survey
EAs: Enumeration Areas
HF: Health Facility
IQR: Interquartile Range
MHR: Median Hazard Ratio
WHO: World Health Organization

## References

[1] Targets SDGH. Ensure healthy lives and promote well-being for all ages. 2016 10.18356/bd21ed60-en

[2] Central Statistical Agency (CSA) [Ethiopia] 2016 and ICF. Ethiopia Demographic and Health Survey 2016. Addis Ababa, Ethiopia, and Rockville, Maryland,; 2016. https://dhsprogram.com/pubs/pdf/FR328/FR328.pdf.

[3] Jordan RG cCndy LF. Prenatal Care and Postnatal Care. 2nd ed. P- 1036.

[4] Yallew MBG and WW. Early Initiation of Antenatal Care and Factors Associated with Early Antenatal Care Initiation at Health Facilities in Southern Ethiopia. Hindawi. 2017;2017: 1–6. 10.1155/2017/1624245 Research

[5] Marshall Makate CM. Prenatal care utilization in Zimbabwe: Examining the role of community-level factors. J Epidemiol Glob Health. 2017;7: 255–262. 10.1016/j.jegh.2017.08.005 29110866 PMC7384567

[6] Who/MSM/2015. Antenatal care and maternal health:How effective is it? Roony C, editor. London; 2015.

[7] Abejirinde Ibukun-Oluwa Omolade, Douwes Renate, Bardají Azucena, Abugnaba-Abanga Rudolf, Zweekhorst Marjolein, et al Pregnant women’s experiences with an integrated diagnostic and decision support device for antenatal care in Ghana. BMC Pregnancy Childbirth. 2018;18: 1–11. 10.1186/s12884-017-1633-9 29871596 PMC5989381

[8] Stacey T, Thompson JMD, Mitchell EA, Zuccollo JM, Ekeroma AJ, McCowan LME. Antenatal care, identification of suboptimal fetal growth and risk of late stillbirth: Findings from the Auckland Stillbirth Study. Aust New Zeal J Obstet Gynaecol. 2012;52: 242–247. 10.1111/j.1479-828X.2011.01406.x 22276935

[9] Turyasiima M, Tugume R, Openy A, Ahairwomugisha E, Opio R, Ntunguka M, et al Determinants of first antenatal care visit by pregnant women at community based education, research and service sites in northern uganda. East Afr Med J. 2015;91: 317–322. PMC4667733 26640281

[10] Gebremeskel F, Dibaba Y, Admassu B. Timing of First Antenatal Care Attendance and Associated Factors among Pregnant Women in Arba Minch Town and Arba Minch District, Gamo Gofa Zone, South Ethiopia. Hindawi Publ Corp J Environ Public Heal. 2015;2015: 1–7. 10.1155/2015/971506 26543485 PMC4620253

[11] Girum T. Assessment of Timing of First Antenatal Care Visit and Associated Factors Among Pregnant Women Attending Antenatal Care in Dilla Town Governmental Health Institutions, Southern Ethiopia. Altern Integr Med. 2016;5: 1–5. 10.4172/2327-5162.1000220

[12] Gudayu Temesgen Worku1 SMW and AAA. Timing and factors associated with first antenatal care booking among pregnant mothers in Gondar. BMC Pregnancy Childbirth. 2014;14: 1–7. Available: http://www.biomedcentral.com/1471-2393/14/287%0A 25154737 10.1186/1471-2393-14-287 PMC4152591

[13] Gidey G, Hailu B, Nigus K, Hailu T, Woldegebriel G, Gerensea H. Timing of first focused antenatal care booking and associated factors among pregnant mothers who attend antenatal care in Central Zone, Tigray, Ethiopia. BMC Res Notes. 2017;10: 1–6. 10.1186/s13104-016-2345-3 29162155 PMC5699019

[14] Utuk NM, Ekanem A, Abasiattai AM. Timing and reasons for antenatal care booking among women in a tertiary health care center in Southern Nigeria. Int J Reprod Contraception, Obstet Gynecol. 2017;6: 3731–3736.

[15] Tekelab Tesfalidet, Chojenta Catherine, Roger Smith DL. Factors affecting utilization of antenatal care in Ethiopia: A systematic review and meta- analysis. PLoS One. 2019;14: 1–24. 10.1371/journal.pone.0214848 30973889 PMC6459485

[16] Atuhaire S, Mugisha JF. Determinants of antenatal care visits and their impact on the choice of birthplace among mothers in Uganda: a systematic review. Obstet Gynecol Int J. 2020;11: 77–81. 10.15406/ogij.2020.11.00492

[17] Paudel YR, Mehata TJ and S. Timing of First Antenatal Care (ANC) and Inequalities in Early Initiation of ANC in Nepal. Front public Heal. 2017;5: 1–6. 10.3389/fpubh.2017.00242 28955707 PMC5600995

[18] Ogbo FA, Dhami MV, Ude EM, Senanayake P, Osuagwu UL, Awosemo AO. Enablers and Barriers to the Utilization of Antenatal Care Services in India. Int J Environ Res Public Heal 2019,. 2019;16: 1–14. Available: www.mdpi.com/journal/ijerph 10.3390/ijerph16173152 31470550 PMC6747369

[19] Obiyan MO, Kumar A. Socioeconomic Inequalities in the Use of Maternal Health Care Services in Nigeria: Trends Between 1990 and 2008. SAGE Open. 2015; 1–11. 10.1177/2158244015614070

[20] Ewunetie AA, Munea AM, Meselu BT, Simeneh MM, Meteku BT. DELAY on first antenatal care visit and its associated factors among pregnant women in public health facilities of Debre Markos. 2018;18: 1–8. 10.1186/s12884-018-1748-7 29769122 PMC5956942

[21] Abuka T, Birhanu AA and B. Assessment of Timing of First Antenatal Care Booking and Associated Factors among Pregnant Women who attend Antenatal Care at Health Facilities in Dilla town, Gedeo Zone, Southern Nations, Nationalities. J Preg Child Heal 3258. 2016;3: 1–7. 10.4172/2376-127X.1000258

[22] Dulla D, Daka D, Wakgari N. Antenatal Care Utilization and Its Associated Factors among Pregnant Women in Boricha District, Southern Ethiopia. Nurs Heal Care Divers. 2017;14: 76–84.

[23] Shibre G, Mekonnen W. Socio-economic inequalities in ANC attendance among mothers who gave birth in the past 12 months in Debre Brehan town and surrounding rural areas, North East Ethiopia: a community-based survey. Reprod Health. 2019;16: 1–14. 10.1186/s12978-018-0662-9 31286965 PMC6615209

[24] Muhwava LS, Morojele N, London L. Psychosocial factors associated with early initiation and frequency of antenatal care (ANC) visits in a rural and urban setting in South Africa: a cross-sectional survey. BMC Pregnancy Childbirth. 2016;16: 1–9. 10.1186/s12884-015-0735-5 26810320 PMC4727269

[25] Ejeta E, Dabsu R, Zewdie O, Merdassa E. Factors determining late antenatal care booking and the content of care among pregnant mother attending antenatal care services in east wollega administrative zone, west Ethiopia. Pan Afr Med J. 2017;27: 1–7. 10.11604/pamj.2017.27.184.10926 28904711 PMC5579454

[26] Hanna G, Gulema H, Berhane Y. Timing of First Antenatal Care Visit and its Associated Factors among Pregnant Women Attending Public Health Facilities in Addis. 2017;27: 1–8. 10.4314/ejhs.v27i2.6 PMC5440828 28579709

[27] Terefe W. Distance from health facility and mothers ‘ perception of quality related to skilled delivery service utilization in northern Ethiopia. Int J Women’s Heal. 2017;7: 749–756. 10.2147/IJWH.S140366 PMC5633329 29042819

[28] Tanou M, Kamiya Y. Assessing the impact of geographical access to health facilities on maternal healthcare utilization: evidence from the Burkina Faso demographic and health survey 2010. BMC Public Health. 2019;19: 1–8. 10.1186/s12889-018-6343-3 31248393 PMC6598277

[29] Tripathi V, Singh R. Regional differences in usage of antenatal care and safe delivery services in Indonesia: fi ndings from a nationally representative survey. BMJ. 2017;7: 1–14. 10.1136/bmjopen-2016-013408 28159851 PMC5293995

[30] Ponna SN, Upadrasta VP, Geddam JJB, Dudala SR, Sadasivuni R, Bathina H. Regional variation in utilization of Antenatal care services in the state of Andhra Pradesh. J Fam Med Prim Care. 2017;6: 231–9. 10.4103/2249-4863.220024 29302523 PMC5749062

[31] Dibaba Y, Id W, Gurmu E, Tilahun T, Bangha M. Contextual influences on the choice of long- acting reversible and permanent contraception in Ethiopia: A multilevel analysis. PLoS One. 2019;14: 1–17. 10.1371/journal.pone.0209602 PMC6334991 30650085

[32] Ministry of Health. Ethiopia. National Guideline for Family Planning Services in Ethiopia. 2019.

[33] Collett David. Modelling Survival Data in Medical Research Third edit. Francesca Dominici, Harvard School of Public Health, USA Julian J. Faraway, University of Bath, UK Martin Tanner, Northwestern University, USA Jim Zidek, University of British Columbia C, editor. london: CRC Press Taylor & Francis Group; 2015.

[34] Lincetto O, Mothebesoane-anoh S, Gomez P, Munjanja S. Antenatal Care.

[35] World Health Organization (WHO). WHO recommendations on antenatal care for a positive pregnancy experience. 2019 pp. 1–172. 28079998

[36] Mamo A, Morankar S, Asfaw S, Bergen N, Kulkarni MA, Abebe L, et al How do community health actors explain their roles? Exploring the roles of community health actors in promoting maternal health services in rural Ethiopia. BMC Health Serv Res. 2019;19: 1–12. 10.1186/s12913-018-3827-x 31638983 PMC6805355

[37] Roy R, Vernekar M. Feto-maternal outcome in grand multipara. Int J Reprod Contraception, Obstet Gynecol. 2017;6: 2846 10.18203/2320-1770.ijrcog20172562

[38] Muniro Z, Tarimo CS, Mahande MJ, Maro E, McHome B. Grand multiparity as a predictor of adverse pregnancy outcome among women who delivered at a tertiary hospital in Northern Tanzania. BMC Pregnancy Childbirth. 2019;19: 1–8. 10.1186/s12884-018-2145-y 31266457 PMC6604326

[39] Mezmur M, Navaneetham K, Letamo G, Bariagaber H. Socioeconomic inequalities in the uptake of maternal healthcare services in Ethiopia. BMC Heal servies Res. 2017;17: 13–17. 10.1186/s12913-017-2298-9 28532407 PMC5441003

[40] GrilliR, RamsayC, MinozziS. Mass media interventions: effects on health services utilisation. Cochrane Database Syst Rev. 2002 10.1002/14651858.CD000389 11869574

[41] Fatema K, Lariscy JT. Mass media exposure and maternal healthcare utilization in South Asia. SSM—Popul Heal. 2020;11: 1–10. 10.1016/j.ssmph.2020.100614 32596437 PMC7306581

